# State-of-the-Art Molecular Biophysics in Russia

**DOI:** 10.3390/ijms25073565

**Published:** 2024-03-22

**Authors:** Oxana V. Galzitskaya

**Affiliations:** 1 Institute of Protein Research, Russian Academy of Sciences, 142290 Pushchino, Russia; ogalzit@vega.protres.ru; 2 Institute of Theoretical and Experimental Biophysics, Russian Academy of Sciences, 142290 Pushchino, Russia

Thirty years ago, scientists’ attention was focused on studying individual molecules, as well as their structure and function. However, in recent years, research has shifted towards the analysis of protein complexes. Firstly, it is crucial to understand how the functioning of protein complexes is regulated, as this affects the binding strength of individual molecules within such complexes. Particular attention is paid to protein complexes, for which structural data are difficult to obtain due to their unusual mobility (flexibility) and their tendency to form highly ordered oligomeric structures [1]. Meanwhile, technology for determining the functional activity of single molecules of enzymes based on nanopores is under continuous development. Natural nanopores have established uses in research involving single molecules of enzymes. Moreover, approaches using artificial solid-state nanopores are also promising, but studies on this technique are currently insufficient. Recently, researchers have demonstrated the use of an approach to study the enzymatic activity of a single molecule of horseradish peroxidase with a solid-state nanopore [2].

Severe acute respiratory syndrome coronavirus 2 (SARS-CoV-2) caused a global pandemic in 2020, resulting in significant challenges for humanity. Many laboratories focused their efforts, first and foremost, on developing a vaccine, and then on finding targets to combat the coronavirus. Due to time constraints, the effectiveness of existing drugs was reconsidered, as this was more efficient than creating entirely new medications. In parallel, efforts were made to create theoretical models for both the growth and spread of infections and the body’s response to the effects of different antibody vaccines. This is because the possibility of antibody-dependent enhancement (ADE) of infection upon vaccination remains poorly understood. The aim of the work was to theoretically determine the conditions leading to the occurrence of ADE in COVID-19 [3]. How SARS-CoV-2 infection leads to an inflammatory response in various types of cells, including those lacking angiotensin-converting enzyme 2 (ACE2) receptors needed for infection, remains a key question in this field [4]. Interestingly, machine learning analysis of the SARS-CoV-2 proteome identified amino acid patterns that mimic host antimicrobial peptides, particularly human cathelicidin LL-37, which may enhance inflammation. The SARS-CoV-2 proteome is enriched in such AMPs compared to low-pathogenic coronaviruses [4]. It transpired that peptides from proteins can not only act as AMPs [5], but can also regulate the activity of receptors. Thus, two peptides from the protein Tag7 were identified and were found to interact differently with the TNFR1 receptor [6]. If the first peptide can activate the receptor together with Hsp70, then the second peptide can inhibit signal transmission through this receptor. Such peptides can be used in the treatment of autoimmune and cancer diseases.

SARS-CoV-2 proteome includes 16 non-structural proteins, 9 accessory proteins, and 4 major structural proteins: the spike (S) protein, the membrane protein, the envelope protein, and the nucleocapsid protein [7]. Each virus particle of SARS-CoV-2 has 26 ± 15 spikes randomly distributed on the virion [8], which is 10 times fewer than the influenza virus [9]. Since the emergence of the original variant in Wuhan in 2019, many different variants of SARS-CoV-2 have been described and characterized, varying in transmissibility and pathogenicity in the human population, although the molecular basis of this difference remains controversial. Specifically, the Omicron variant is known for its contagiousness, transmissibility, and lower pathogenicity (mortality). This is largely attributed to amino acid substitutions on the surface of the Spike protein, which interact with the ACE2 receptor; these can facilitate the penetration of the virus into the cell or help it to evade the immune response. Mutations in this strain result in increased amyloidogenicity for the Omicron strain in the ACE2 receptor binding regions, resulting in an increase in the strength of this interaction for the Omicron BA.1 RBD compared to the Wuhan-Hu-1 or Delta RBD, and this effect is more pronounced at pH 5 [10]. This result is associated with Omicron variants’ increased ability to spread through the population. It has been suggested that the more positively charged surface of the Omicron RBD variant may facilitate its distribution in the upper respiratory tract, but not in the lower respiratory tract, where pH estimates vary [10].

It should be noted that Cui et al. identified pH-dependent structural changes in the S-protein, which affect the RBD domain [11]. In Figure S2, they show the structures at different pH. Electrostatic RBD surfaces of different variants are shown in Figures 4I and S6 in the paper [12], showing the difference between the variants. Moreover, an electrostatic map of the external surface of SARS-CoV-2 was obtained in the paper [13].

One way to end the Coronavirus impasse is to review existing medications. More than 800 papers have been published regarding in silico and in vitro screening of anti-SARS-CoV-2 activity of known drugs [14,15].

Recent work has revealed that S-acylation of the SARS-CoV-2 spike is required for virus infectivity [16,17]. Therefore, to combat SARS-CoV-2, it is proposed that we should block DHHC20, an acylating S protein [18,19], since S-acylation of SARS-CoV-2 spike protein induces lipid reorganization. Cys residues from a cytoplasmic tail of S2 subunit are sites for post-translational modification by S-acylation [16,20,21], a reaction that is mediated by host cell S-acyltransferases belonging to the zinc finger Asp-His-His-Cys domain-containing (ZDHHC) family of transmembrane enzymes. In [22], the authors attempt to find selective inhibitors targeting ZDHHC20 acyltransferases involved in spike S-acylation [22]. This is just the beginning; future research will aim to further our understanding of protein S-acylation, as this process is critical for the replication cycle of other pathogenic viruses.

Research examining the functioning of the complexes can be considered one of the most advanced approaches in the field of structure-based rational drug discovery and will significantly reduce the time and money spent searching for new drugs. Nowadays, not a single medicine is created without the use of computer calculations [23].
